# The Role of TGF-β Signaling in Lung Cancer Associated with Idiopathic Pulmonary Fibrosis

**DOI:** 10.3390/ijms19113611

**Published:** 2018-11-15

**Authors:** Akira Saito, Masafumi Horie, Patrick Micke, Takahide Nagase

**Affiliations:** 1 Department of Respiratory Medicine, Graduate School of Medicine, The University of Tokyo, 7-3-1 Hongo, Bunkyo-ku, Tokyo 113-0033, Japan; mhorie-tky@umin.ac.jp (M.H.); takahide-tky@umin.ac.jp (T.N.); 2 Division for Health Service Promotion, The University of Tokyo, 7-3-1 Hongo, Bunkyo-ku, Tokyo 113-0033, Japan; 3 Hastings Center for Pulmonary Research, Division of Pulmonary, Critical Care and Sleep Medicine, Department of Medicine, Keck School of Medicine, University of Southern California, Los Angeles, CA 90033, USA; 4 Department of Immunology, Genetics and Pathology, Uppsala University, SE-75185 Uppsala, Sweden; patrick.micke@igp.uu.se

**Keywords:** idiopathic pulmonary fibrosis, non-small cell lung cancer, transforming growth factor-β, extracellular matrix, tumor microenvironment, usual interstitial pneumonia

## Abstract

Idiopathic pulmonary fibrosis (IPF) is a progressive fibrotic lung disease of unknown etiology and dismal prognosis. IPF patients are known to have an increased risk of lung cancer and careful decision-making is required for the treatment of lung cancer associated with IPF. Transforming growth factor (TGF)-β signaling plays a central role in tissue fibrosis and tumorigenesis. TGF-β-mediated pathological changes that occur in IPF lung tissue may promote the process of field cancerization and provide the microenvironment favorable to cancer initiation and progression. This review summarizes the current knowledge related to IPF pathogenesis and explores the molecular mechanisms that underlie the occurrence of lung cancer in the background of IPF, with an emphasis on the multifaceted effects of TGF-β signaling.

## 1. Introduction

Idiopathic pulmonary fibrosis (IPF) is a progressive and fatal lung disease of unknown etiology, and is the most common subtype of idiopathic interstitial pneumonia. The diagnosis of IPF is based on radiological and/or pathological findings, indicative of the morphological pattern of usual interstitial pneumonia (UIP) [1]. The incidence of IPF is estimated to be 3–9 cases per 100,000 person-years [2]. IPF is comprised of sporadic and familial cases, suggesting that genetic factors of variable degree are involved in its pathogenesis [3]. Prognosis for IPF patients is poor with a median survival time of only 2–4 years; however, the clinical course of IPF is highly variable and some patients can survive for long periods of time [4]. Despite recent advances in anti-fibrotic therapies, IPF remains an incurable disease [1,3,4].

Lung cancer is a major comorbidity in IPF patients [5]. A number of studies have demonstrated a strong association between IPF and lung cancer, and epidemiological studies have shown that IPF itself is an independent risk factor for lung cancer [5,6,7]. The reported prevalence of lung cancer among IPF patients is 3–48% [6]. This variance appears to be due to the differences in study populations. A smaller Japanese study calculated the cumulative incidence rates of lung cancer for IPF patients to be 3.3%, 15.4%, and 54.7% at 1, 5, and 10 years, respectively [8]. The survival time of IPF patients with lung cancer is shorter than those without lung cancer [9].

The strong association between IPF and lung cancer indicates that the molecular changes underlying IPF pathogenesis have tumor-promoting effects. Indeed, similar mechanisms have been suggested to be involved in both IPF and lung cancer, including telomere abnormalities, alterations in alveolar epithelial cell features, epithelial–mesenchymal transition (EMT), sustained fibroblast activation, and extracellular matrix (ECM) remodeling [3,4,10,11]. Notably, the anti-fibrotic drug for the treatment of IPF, nintedanib, is also approved as a therapeutic agent for non-small cell lung cancer [12]. Thus, the pathogenic overlap of both diseases may help to better understand the molecular mechanisms involved in IPF and lung cancer, and ultimately may provide clues in developing therapeutic strategies for both diseases.

Transforming growth factor (TGF)-β is a key player in the process of fibrosis and tumorigenesis. TGF-β, as a pleiotropic factor, regulates a variety of biological processes including embryogenesis, cellular differentiation, organogenesis, and immune responses [13]. Under physiological conditions, TGF-β is necessary for lung morphogenesis and homeostasis while aberrant TGF-β signaling has been shown to be central to pulmonary fibrosis and lung cancer progression [11].

In this review, we integrate the current literature of IPF pathogenesis and its relationship with lung cancer, with an emphasis on TGF-β signaling. First, we present an overview of current knowledge about genetic predispositions to IPF and explore its association with lung carcinogenesis. Furthermore, we summarize the recent reports on the genomic features of lung cancer that occurred in the background of IPF. In the latter part of this review, TGF-β signaling is highlighted as a key molecular link between IPF and lung cancer, and we discuss its multifaceted roles in alveolar epithelial cells, fibroblasts, and immune cells.

## 2. Genetic Predispositions to Pulmonary Fibrosis

Both genetic and environmental factors contribute to the development of IPF. Risk factors for IPF identified through epidemiological studies include ageing, male sex, tobacco smoking, and other environmental exposures [14]. Notably, pulmonary fibrosis tends to occur among smokers in a pedigree of familial pulmonary fibrosis, suggesting that both genetic and environmental factors influence its pathogenesis [15]. Indeed, a significant number of IPF patients are former or current smokers (40–70%) [5,16]. Tobacco smoking is the major risk factor for lung cancer [17], which may confound the association between IPF and lung cancer. However, a population-based cohort study in the United Kingdom demonstrated that the incidence of lung cancer has increased independent of smoking history in IPF patients [5].

In addition to environmental factors, recent genome-wide association studies have identified genetic polymorphisms that are related to an increased risk of IPF; those reported by multiple studies include the regions 5p15 (*TERT*) and 3q26 (*TERC*) [18,19]. Telomeres are DNA–protein complexes that protect the ends of chromosomes from degradation and serve to maintain genomic integrity. Shortening of telomeres occurs during each cell division and is implicated in cellular ageing [20]. Telomerase is a ribonucleoprotein complex involved in the addition of repetitive DNA sequences to telomeres and is comprised of telomerase reverse transcriptase (*TERT*) and telomerase RNA component (*TERC*).

Besides the telomerase genes, *TERT* and *TERC*, multiple studies have identified genetic polymorphisms in the 11p15 region (*MUC5B* and *TOLLIP*) related to the risk of IPF [21,22,23], and the genotype of *MUC5B* and *TOLLIP* has been shown to be significantly associated with prognosis and therapeutic responses among IPF patients [24,25,26]. It is estimated that the rs35705950 variant in the promoter of *MUC5B* is the strongest risk factor for developing IPF [27]. Mechanistically, it has been proposed that the rs35705950 variant leads to overexpression of mucin 5B, which may cause abnormal mucociliary clearance and altered immune responses, ultimately leading to chronic alveolar epithelial cell damage [28].

Most convincing evidence of genetic predispositions to IPF comes from pedigrees with familial pulmonary fibrosis. Mutations in surfactant protein C (*SFTPC*) and surfactant protein A2 (*SFTPA2*) and their associations with familial pulmonary fibrosis suggest the importance of alveolar epithelial cells in IPF pathogenesis [29,30]. The frequency of *SFTPC* mutations in familial cases of IPF seems variable depending on ethnicity (2–25%) [31]. In correspondence to the association between *TERT* or *TERC* gene polymorphisms and the risk of sporadic IPF, germline *TERT* or *TERC* mutations have been found in 8–15% of familial cases of IPF [32,33,34,35]. Two more genes linked to telomere biology are mutated in a subset of familial cases: regulator of telomere elongation helicase 1 (*RTEL1*) and poly(A)-specific ribonuclease (*PARN*) [36,37].

It is known that mutations in *TERT* and *TERC* are related to telomere shortening as a result of impaired telomerase catalytic activity. *RTEL1* is required to prevent catastrophic telomere loss and its mutation causes telomere instability [38]. It has been recently shown that *PARN* is required for post-transcriptional RNA maturation of the *TERC* gene, further supporting the importance of telomere dysfunction in IPF pathogenesis [39].

Even in the absence of mutations in the telomere-related genes, shortening of telomeres in alveolar epithelial cells is commonly found in IPF patients [34]. In alveolar epithelial type II cells, which are regarded as stem cells in the alveoli [40], telomere shortening results in cellular senescence, stem cell failure, and pulmonary fibrosis in a mouse model [41,42]. Thus, telomere dysfunction through gene–environment interactions appears to be one major pathological mechanism of IPF in general.

Besides the regulatory role of *TERT* and *RTEL1* for telomere function, both genes are also required for DNA replication and DNA repair, ensuring genome integrity [43,44]. This function is of particular importance for alveolar epithelial type II cells that are exposed to a myriad of environmental pollutants and carcinogens. Because alveolar epithelial type II cells are considered to be an origin of lung cancer [45], telomere dysfunction and functional abnormalities of *TERT* and *RTEL1* genes might conceptually connect IPF to lung carcinogenesis.

Apart from telomere dysfunction and shortening involved in IPF pathogenesis, mRNA upregulation or amplification of *TERT* and *TERC* genes is frequently found in lung adenocarcinomas and squamous cell carcinomas [46,47]. It is conceivable that *TERT* and *TERC* upregulation or amplification leads to increased telomerase activity, thereby avoiding replicative senescence and gaining growth advantage [48]. However, it remains unexplored whether and how telomerase activity is altered during the process of lung carcinogenesis in IPF patients.

## 3. Genomic Features of Lung Cancer That Occurs in Pulmonary Fibrosis

In recent years, genomic alterations and mutation profiles of lung cancer have been extensively investigated in large cohorts including The Cancer Genome Atlas (TCGA) [46,47]. In general, adenocarcinoma is the most frequent histological subtype of lung cancer (30–40%), followed by squamous cell carcinoma (20–30%) [49]. In contrast, squamous cell carcinoma is the most prevalent subtype (35–40%) of lung cancer that occurs in IPF, and adenocarcinoma accounts for 30–35% of cases [50].

Based on the observation that genomic aberrations and precancerous lesions occur multifocally in a region or an organ, the concept of field cancerization has now been recognized as the priming of normal tissue that precedes the occurrence of cancer [51,52]. A recent report demonstrated that gene expression profiles from bronchial epithelial cells with normal appearance are useful in assessing lung cancer probability [53]. Another report demonstrated that *TP53* mutations associated with tobacco smoking are observed in the lungs prior to the development of clinically detectable cancer [54]. These reports suggest that cancer-prone abnormal cells reside in morphologically non-cancerous lung tissues. In IPF lung tissue, progressive bronchiolar proliferation, termed bronchiolization, and squamous metaplasia are frequently found in the fibrotic area [55]. These metaplastic foci have been demonstrated to be associated with atypia and the occurrence of *TP53* mutations [56,57]. It is tempting to speculate that these lesions serve as precursors to lung cancer, which may provide an explanation for the higher prevalence of squamous cell carcinoma in IPF patients. Moreover, such a scenario seems to fit with the concept of field cancerization.

Recently, genetic and clinicopathological features of 44 lung adenocarcinomas in the background of UIP, which is the morphological characteristic of IPF, have been analyzed in detail [58]. Compared to lung adenocarcinomas without UIP, adenocarcinomas with comorbid UIP were associated with older age, male gender (93.2%), and smoking history (90.9%). Importantly, adenocarcinomas with UIP showed higher frequencies of invasive mucinous-predominant subtype (29.5% vs. 3.9%) and *KRAS* mutations (30.2% vs. 8.5%) while *EGFR* mutations were much less prevalent (2.3% vs. 45.6%) [58]. These observations are consistent with the finding that *KRAS* mutation-positive lung adenocarcinoma is associated with male sex and smoking history [59].

Recently, it has been demonstrated that MUC5B serves as a marker for the non-terminal respiratory unit (TRU) subtype and the invasive mucinous adenocarcinomas associated with *KRAS* mutations [60,61]. As mentioned above, *MUC5B* polymorphism is associated with pulmonary fibrosis [21,22,23], and presumably, mucin 5B is overexpressed in IPF lung tissue. This may be somehow related to the observation that the invasive mucinous-predominant subtype is more frequent in lung adenocarcinomas with UIP [58].

Another study profiled 15 adenocarcinomas and 20 squamous cell carcinomas associated with IPF by sequencing ~500 cancer-related genes [62]. The mean number of somatic mutations was 11.7 per sample with an average of 5.86 mutations/Mb. Interestingly, the frequency of *BRAF* mutations was high (17.1%), while in this study, *KRAS* mutation was not found in any sample [62]. *EGFR* mutation was detected paradoxically in one case of squamous cell carcinoma. This is in contrast with the previous study [58] that showed a higher frequency of *KRAS* mutations in lung adenocarcinomas associated with UIP. Both studies are based on surgically resected lung cancer samples of East Asian populations, in which the frequencies of *KRAS* and *EGFR* mutations are reported to be 8–10% and 40–55%, respectively, in lung adenocarcinomas [59]. Further studies with larger sample size are needed to elucidate oncogenic drivers dominant in lung adenocarcinomas or squamous cell carcinomas that occur in IPF lung tissue.

## 4. Genome-Wide Methylation Profiles of Pulmonary Fibrosis and Lung Cancer

In recent years, genome-wide methylation profiling studies have identified hypermethylated and hypomethylated genes in lung cancer [63]. In general, DNA hypermethylation of tumor suppressor genes is implicated in lung cancer development. For example, promoter hypermethylation of *CDKN2A* and *RASSF1* leads to perturbed cell cycle regulation, which contributes to lung carcinogenesis. Importantly, hypermethylation of these tumor suppressors has been shown to be associated with smoking behavior in lung cancer patients [64].

In addition, global DNA hypomethylation is also a hallmark of cancer, causing aberrant expression of cancer testis antigens and activation of repetitive elements, such as long interspersed nuclear element-1 (LINE-1), across the whole genome [63,65]. This is clinically important, as transactivation of cancer testis antigens has been postulated to enhance the efficacy of immune therapy. Furthermore, it has been suggested that detection of transactivated repetitive elements might be potentially useful for the diagnosis of lung cancer [63].

There have been few studies describing DNA methylation patterns in the lung tissues of IPF patients [66]. A pioneering study of profiling genome-wide methylation in 94 IPF patients and 67 controls identified 2130 differentially methylated regions, and among them, 738 regions were inversely correlated with gene expression changes [67].

Another study identified 402 CpG islands that were differentially methylated in both IPF lung tissues and lung adenocarcinomas compared with control lungs [68]. Interestingly, IPF lung tissue displayed an intermediate methylation profile of these CpG islands, between normal and cancerous tissues. This observation suggests that DNA methylation patterns in IPF lung tissue may constitute precancerous changes in line with the concept of field cancerization. However, in the same study, IPF lung tissue did not show hypomethylation of LINE-1 repetitive elements, one of the methylation patterns frequently found in lung cancer. This observation suggests that lung cancer DNA methylation profiles are, in some aspects, distinct from those of IPF.

## 5. Roles of TGF-β in Alveolar Epithelial Cells

**Figure 1:**
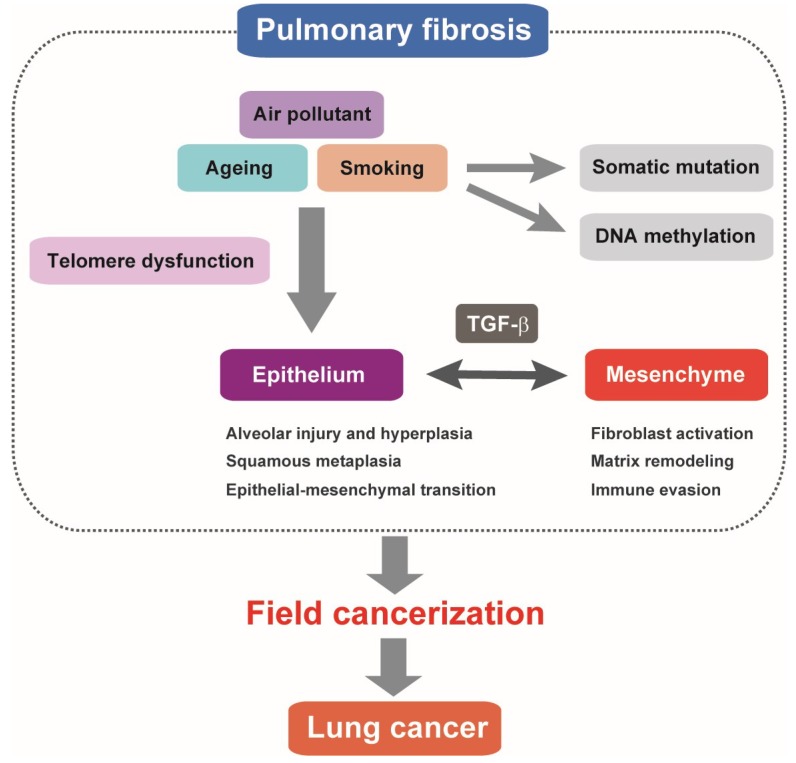
Pulmonary fibrosis as field cancerization. Alveolar epithelial cell injury and regeneration failure associated with telomere dysfunction represent a hallmark of pulmonary fibrosis. Pathologically activated TGF-β signaling is involved in altered alveolar epithelial cell features and dysregulated epithelial–mesenchymal interactions. It is postulated that somatic mutations and DNA methylation changes accumulating in pulmonary fibrosis multifocally predispose to lung cancer. TGF-β-mediated fibrotic and immune-suppressive microenvironment in the lung tissue of pulmonary fibrosis may have tumor-promoting features similar to lung cancer stroma.

In IPF lung tissue, pathological events include alveolar epithelial regenerative failure (repeated epithelial damage and aberrant repair of the injured epithelium) and sustained fibroproliferative reactions (persistent fibroblast activation, excessive ECM deposition, and the resultant structural alterations) [3,4]. Molecular processes of alveolar epithelial cell damage and defective repair comprise oxidative injury, endoplasmic reticulum stress, mitochondrial dysfunction, cellular senescence, and apoptosis [69,70]. Transforming growth factor (TGF)-β is essential for both epithelial and mesenchymal changes (Figure 1). 

Although many other inflammatory signals are involved in IPF pathogenesis, such as interleukin (IL)-1β, IL-13, IL-17, CC chemokine ligand 2 (CCL2), and CXC chemokine ligand 12 (CXCL12) [71], in a clinical setting, anti-inflammatory or immunosuppressive agents, including corticosteroids, did not show clear therapeutic benefits. This supports the hypothesis that IPF is caused by dysregulated epithelial–mesenchymal interactions and is not simply an inflammatory disease.

TGF-β is abundantly expressed in IPF lung tissue, where macrophages and metaplastic alveolar epithelial cells are the major cellular sources of TGF-β [72,73]. In rodent models, ectopic expression of TGF-β in the lungs recapitulates the pathophysiological features of human pulmonary fibrosis, supporting the concept that TGF-β plays a pivotal role in the disease pathogenesis [74,75]. TGF-β induces cytostasis in most epithelial cells and inhibits proliferation of alveolar epithelial cells [76]. Thus, it is conceivable that TGF-β negatively affects epithelial cell regeneration and therefore contributes to IPF pathogenesis.

TGF-β is also known to strongly elicit EMT, a phenotypic change whereby polarized epithelial cells acquire mesenchymal and migratory characteristics [14]. Mechanistically, TGF-β induces the transcriptional repressors, SNAI1, SNAI2, ZEB1, and ZEB2, which subsequently repress cell junctional proteins, in turn, disrupting epithelial cell integrity and apical–basal polarity. Previous studies have demonstrated that TGF-β elicits EMT in alveolar epithelial cells, which may be a part of the altered epithelial features in IPF lung tissue [77]. It remains controversial whether epithelial cells that undergo EMT contribute to mesenchymal cell populations, with conflicting results from studies of cell lineage tracing in mouse models [78].

Single-cell transcriptome analysis is emerging as a powerful method for deciphering heterogeneous cell populations and cellular differentiation processes [79]. Recently, single-cell RNA-sequencing of lung epithelial cells from IPF patients has been performed, which identified three subtypes of epithelial cells associated with IPF. Their transcriptional profiles were distinct from those of alveolar epithelial type II cells, and showed features of conducting airway basal cells or goblet cells, supporting the concept of bronchiolization. Notably, mesenchymal markers and EMT-related molecules were induced in “basal cell” or “indeterminate” subtypes of IPF-derived epithelial cells, indicating the involvement of EMT. In line with this finding, TGF-β signaling pathway genes showed higher expression levels in IPF-derived epithelial cells and pathway analysis revealed aberrant activation of TGF-β signaling [79].

In contrast to normal epithelial cells with organized cell junction and polarity, most cultured cancer cells lack cell polarity and exist in an intermediate cellular status between epithelial and mesenchymal cells, which is termed partial EMT. Progression and completion of EMT processes in cancer cells are associated with the acquisition of malignant cell features including stem cell traits, resistance to apoptosis, cell invasion, and drug resistance [80].

Taken together, it can be postulated that TGF-β-mediated EMT in IPF lung tissue might contribute to malignant transformation. However, further pathological evaluations are required to determine whether EMT is clinically relevant and significantly involved in the fibrotic process of IPF. Moreover, it also needs to be determined whether EMT-related molecular changes are associated with the morphological changes found in IPF, such as alveolar epithelial cell hyperplasia and squamous metaplasia.

## 6. TGF-β-Mediated Fibroproliferative Reactions in Pulmonary Fibrosis and Lung Cancer

In addition to aberrations in epithelial cells, pathological activation of fibroblasts and excessive ECM accumulation constitute a major hallmark of IPF, leading to irreversible structural alterations and tissue stiffening in the lungs. These changes functionally cause lung volume loss and a reduced vital capacity. The fibrotic thickening of the alveolar wall impairs gas exchange with decreased diffusing capacity and ultimately leads to arterial hypoxemia [3,4].

Activated lung fibroblasts, which conceivably overlap with α-smooth muscle actin (α-SMA)-positive myofibroblasts, are responsible for producing ECM components, such as collagen and laminin [81]. TGF-β potently enhances differentiation from fibroblast to myofibroblast, and promotes the expression of ECM components [11]. Moreover, TGF-β induces the expression of integrins, matrix metalloproteinases, protease inhibitors, and regulators of small GTPases, all of which contribute to tissue remodeling and cell–ECM interactions [82]. TGF-β also stimulates the production of fibrogenic or angiogenic growth factors such as connective tissue growth factor (CTGF), platelet-derived growth factor (PDGF), and vascular endothelial cell growth factor (VEGF).

Corresponding to mesenchymal tissue remodeling in IPF, TGF-β is also central in the development of tumor stroma, which is composed of cancer-associated fibroblasts (CAFs), immune cells, and the ECM. In analogy to IPF, CAFs are α-SMA-positive myofibroblasts activated by TGF-β, and have common features with the myofibroblasts observed in IPF lung tissue [83]. Notably, gene expression profiling analyses have demonstrated that CAFs isolated from lung cancer tissues exhibit increased expression of TGF-β signaling-related genes [84,85].

**Figure 2:**
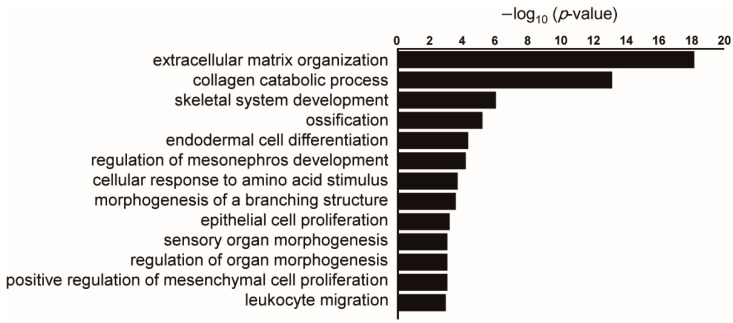
Pathway analysis of genes commonly upregulated in lung cancer stroma and interstitial pneumonia compared to normal lung tissues. Public datasets of gene expression profiling in lung cancer stroma (GSE22863) and interstitial pneumonia (GSE47460) were compared. When the top 1000 significantly upregulated genes in each dataset were compared, 101 genes were common between these two datasets. Enriched gene ontology terms for biological process were sorted by –log10 (*p*-value). When there are several similar terms such as “extracellular matrix organization” and “extracellular structure organization”, one term with a lower p-value was selected.

To compare gene expression profiles from lung cancer stroma samples and the lung tissues of interstitial pneumonia including IPF, we utilized publicly available datasets. The dataset GSE22863 comprises gene expression profiles from normal lung parenchyma and lung tumor stroma while the dataset GSE47460 includes gene expression profiles from control lung tissues and different interstitial lung diseases including IPF. We found that about 10% of genes significantly upregulated in lung cancer stroma were also enriched in lung tissues of interstitial lung diseases compared to normal lung tissues. These commonly upregulated genes included ECM components (COL1A2, COL3A1, and COL5A2) and matrix metalloproteinases (MMP9 and MMP11). Importantly, inferred biological processes of these commonly upregulated genes were mainly related to ECM remodeling and collagen metabolism (Figure 2). These findings support the idea that cancer stroma exhibits molecular features similar to those of interstitial pneumonia or IPF.

The mesenchymal changes in IPF lung tissue, most likely mediated by TGF-β, also influence the repair process of the alveolar epithelium by inducing developmental signals. Particularly, activated fibroblasts produce growth factors for the epithelium such as hepatocyte growth factor (HGF). In IPF patients, HGF levels in serum and bronchoalveolar lavage fluid are significantly higher than in normal controls [86]. Considering the importance of the HGF/c-Met pathway for lung cancer progression [87], an abundance of growth factors in the milieu of IPF lung tissue may facilitate the development of cancer in IPF patients.

Finally, TGF-β signaling is essential in the regulation of immune responses. Its role is evidenced by the systemic inflammation observed in TGF-β1-deficient mice [88,89]. Of particular importance is its involvement in T cell homeostasis and immune-suppressive effects [90]. Cancer progression is dependent on the evasion of immunosurveillance, and immune suppression by TGF-β is an emerging mechanism in tumorigenesis [91,92]. Furthermore, accumulating evidence supports the notion that TGF-β mediates tumor-promoting properties of tumor-associated macrophages and myeloid-derived suppressor cells [93].

A previous study performed immunohistochemistry for immune cell markers on IPF lung tissue and characterized topological patterns of immune cell infiltration. The fibrotic regions had much fewer lymphocytes, monocytes, and macrophages compared to the epithelial dominant areas. This observation suggests that fibrotic lesions in IPF lung tissue might be privileged to escape from immune surveillance. It has been reported that lung cancer might preferentially originate from the fibrotic area as felicitously termed “scar-cinoma” [94]. In support of this notion, lung cancers associated with IPF are frequently found in the lower lobe and peripheral portion where fibrotic changes predominantly occur.

Collectively, IPF lung tissue with abundant TGF-β could represent a microenvironment in which anti-cancer immune surveillance is disrupted, which is a condition favorable to cancer initiation and progression. However, the similarities and differences of immune landscape between IPF and lung cancer tissues remain largely unknown.

## 7. Clinical Implications and Future Perspectives

The diagnosis of lung cancer in patients with IPF is difficult because radiological findings of cancerous lesions often appear indistinct from the fibrotic or inflammatory changes associated with the histological pattern of UIP. In a clinical setting, special attention is required in therapeutic decision-making for lung cancers complicated with IPF, and standard treatment guidelines for lung cancer are not applicable to patients with IPF. Radiation therapy, whether curative or palliative, is not recommended for IPF patients because of the risk of acute exacerbation. EGFR-tyrosine kinase inhibitors are known to induce fatal acute interstitial pneumonia with a higher frequency among lung cancer patients with prior pulmonary fibrosis (mostly IPF) [95]. For early-stage resectable lung cancers, acute exacerbation of interstitial pneumonia following surgery is often fatal, and it is difficult to estimate the risk of this occurring. It has been reported that acute exacerbation following thoracic surgery may occur in 9% of cases, with a mortality rate of more than 40% [96,97].

Recently, nintedanib and pirfenidone have both been approved as treatment options for IPF [3,4]. Nintedanib is a multi-kinase inhibitor that targets VEGF receptors, PDGF receptors, and fibroblast growth factor receptors. Pirfenidone exerts its effect through downregulation of TGF-β, suppression of fibroblast proliferation, and inhibition of collagen synthesis. These agents slow disease progression [98,99], and recent meta-analyses have demonstrated that these agents have a beneficial impact on survival [100,101].

Importantly, nintedanib has been shown to be effective as a therapeutic agent for non-small cell lung cancer [12]. These clinical observations further suggest the close relationship between IPF and lung cancer at the molecular level.

An obvious question is whether a significant amount of somatic mutations are accumulating in the fibrotic lesion of IPF lung tissue. If so, are they related to the degree of fibrotic changes or precancerous stages? Transcriptome and methylation profiles of lung cancer in IPF patients also remain to be elucidated. Recent studies have suggested that lung cancers of each histological subtype can be further subclassified based on gene expression and methylation patterns, which is related to different responses to cytotoxic agents, molecular targeted therapies, and immune checkpoint inhibitors [102,103]. Given that the mutation burden of lung cancer complicated with IPF is relatively high, as suggested by a recent report [62], immune checkpoint inhibitors might be beneficial. However, interstitial lung disease is one of the immune-related adverse events, which may exacerbate IPF [104], and therefore immune checkpoint blockade should be considered with caution.

The genomic changes that occur during the evolution of lung cancer in IPF lung tissue are still fragmentarily understood, and further studies are necessary to dissect the contributions of telomere dysfunction, smoking, epigenetic changes, and somatic mutations to cancer initiation and progression in IPF patients. In summary, a better understanding of IPF pathogenesis and its association with cancer will aid in the development of more refined diagnostic and therapeutic strategies for both IPF and lung cancer.
